# Noninvasive Encapsulated Follicular Variant of Papillary Thyroid Cancer: Clinical Lessons from a Community-Based Endocrine Surgical Practice

**DOI:** 10.1155/2017/4689465

**Published:** 2017-04-13

**Authors:** Allan Golding, Dana Shively, David N. Bimston, R. Mack Harrell

**Affiliations:** ^1^Department of Endocrine Surgery, Memorial Hospital System, Hollywood, FL, USA; ^2^The Department of Undergraduate Studies, The University of Florida, Gainesville, FL, USA

## Abstract

*Objective*. Retrospective studies have found that noninvasive encapsulated follicular variant of papillary thyroid cancer (EFVPTC) exhibits highly indolent clinical behavior. We studied the clinical features of our patients with noninvasive EFVPTC tumors culled from a community endocrine surgical practice registry over the past four years.* Methods*. We interrogated the Memorial Center for Integrative Endocrine Surgery (MCIES) Registry for all recorded encapsulated follicular variant of papillary cancer pathologic diagnoses. We identified a subgroup of patients without capsular or vascular invasion and studied their clinical characteristics.* Results*. Thirty-seven patients met inclusion and exclusion criteria. The typical patient was young and female. Nodules averaged 3.1 cm in greatest dimension by ultrasound evaluation. Thirteen patients were found to have synchronous malignancies elsewhere in the thyroid (35%). At the time of this writing, we have not seen a clinical recurrence in any of our 37 noninvasive EFVPTC patients.* Conclusions*. Early clinical follow-up data suggests that the majority of noninvasive EFVPTC tumors exhibit indolent behavior, but clinical decision-making with regard to completion thyroidectomy, central lymph node dissection, and adjunctive radioiodine therapy often depends on the amount and type of synchronous thyroid cancer detected elsewhere in the thyroid gland and the central neck.

## 1. Introduction

The incidence of thyroid cancer diagnoses has tripled over the past 30 years [1]. Encapsulated follicular variant of thyroid cancer (EFVPTC) now represents up to 20% of all thyroid cancers reported [1–3]. Recently the Endocrine Pathology Society Working Group has proposed a revised nomenclature that eliminates the word “cancer” from the diagnostic title of a subset of noninvasive EFVPTCs by creating the new classification “noninvasive follicular tumors with papillary-like nuclear features” (NIFTP). To support the new nomenclature, the working group validated the name change with a review of 210 retrospectively collected EFVPTC-diagnosed patients, reclassifying 103 of them as NIFTP patients, in whom not one had evidence for thyroid cancer recurrence over 10–26 years of follow-up [4]. The clinical observation that a subset of noninvasive EFVPTCs may behave in a benign fashion is not new. In 2006, Liu et al. reported on 42 noninvasive EFVPTCs, 31 of which had been treated with lobectomy alone. After a median follow-up of 11 years, none developed lymph node metastases or recurrences [5]. In 2011, Daniels suggested that it might be possible to reclassify some FVPTCs as benign neoplasms [6].

In light of recent important changes in thyroid pathological classification, we sought to retrospectively evaluate our community endocrine surgical experience with noninvasive EFVPTCs over the past 45 months. Based on our clinical, ultrasound, and fine needle aspiration biopsy cytology data, we propose an algorithm for surgical management in suspected noninvasive EFVPTC patients.

## 2. Methods

We queried the Memorial Center for Endocrine Surgery Registry for fully encapsulated follicular variant of papillary cancer diagnoses from January 2012 through September 2016. All these tumors were removed by a single surgeon, DNB, and had pathologic diagnoses rendered by a single pathology group serving our six-hospital consortium with a patient referral base encompassing South Florida and the Caribbean (Memorial Health Systems). Neck ultrasound evaluation was performed by AG or RMH for 36 out of 37 patients (97% of patients). Thyroid fine needle aspiration (FNA) biopsies were performed by AG or RMH in 15 thyroid nodules (40.5%) with 15 nodule FNAs (40.5%) performed by outside physicians. Seven nodules (19%) were not biopsied prior to removal. Cytology was read by Thyroid Cytopathology Partners of Austin, Texas, in 51% of cases and a combination of CBL and other community reference laboratory cytopathologists in 49%.

Tumors in which the pathologist identified any vascular invasion or any capsular discontinuity or invasion were excluded from consideration in this report. Isolated subcentimeter encapsulated follicular variant of papillary tumors (EFVPTC) that were incidentally noted by the pathologist following surgery for another indication (symptomatic goiter, other larger non-EFVPTC tumors) were excluded. In addition, tumors without ultrasound data were excluded (one patient).

We systematically reviewed electronic medical records on each of the subjects to confirm that ultrasound, cytology, and pathology reports were concordant on a nodule-by-nodule basis.

## 3. Results

Twelve hundred and ten unique patients underwent thyroid surgery at the Memorial Center for Integrative Endocrine Surgery from January 2013 through September of 2016, of which 796 demonstrated nonincidental thyroid malignancies. Of these, 413 (52%) were follicular variant of papillary cancers (FVPTC). Eighty-eight (21%) of the FVPTC tumors were fully encapsulated (EFVPTC) and 50 (12%) were fully encapsulated with no evidence of capsular or vascular invasion and were considered noninvasive EFVPTC. In these fifty patients, neither capsular invasion, vascular invasion, nor papillary architectural features were identified in the primary tumor pathology report. Twelve of these tumors were excluded because they were subcentimeter incidental lesions discovered by our pathologists in thyroid glands removed for other reasons (symptomatic goiter or larger non-EFVPTC tumors). One tumor was excluded due to the absence of ultrasound data. Thus 37 out of 796 (4.6%) of our resected thyroid malignancies were found to meet our inclusion criteria as noninvasive encapsulated follicular variant of papillary thyroid cancers removed by our surgeon from 1/2013 to 9/2016. Thirty-seven tumors of interest were found in 37 patients.

In our practice, the typical noninvasive EFVPTC tumor patient had an average age of 44 years. Twenty-nine of the 37 patients were female (78%).

On neck ultrasound, noninvasive EFVPTC nodules had a mean diameter of 3.1 cm in greatest dimension (range 0.8 cm–6.9 cm with a median size of 3 cm). Nodules were evenly distributed throughout the thyroid with 18 (49%) in the right lobe, 17 (46%) in the left lobe, and 2 (5%) in the isthmus. Echogenicity was variable, with 9 (24%) read as hypoechoic, 21 (57%) read as isoechoic, and 7 (19%) described as heteroechoic on ultrasound. Thirty-three nodules (89%) were solid and 4 (11%) were described as partially cystic. Borders were sharp and well defined in 34 nodules (92%). Three nodules had hypoechoic halos (8%). Thirty-six nodules (97%) lacked intranodular calcifications, whereas intranodular arcuate calcification was described in one nodule. Twelve nodules (32%) demonstrated grade 1 vascularity as assessed by Power Doppler, 11 nodules had grade 2 vascularity (30%), and 14 nodules had grade 3 vascularity (38%). No nodules had grade 4 vascularity. The mean vascularity grade was 2.1.

Thirty patients out of 37 (81%) underwent fine needle aspiration biopsy prior to surgery. Seven patients were referred directly to surgery based on sonographic findings alone. Eight nodules (27%) yielded benign cytological results (Bethesda 2), 14 (47%) nodules demonstrated atypia of uncertain significance (Bethesda 3), and 8 (27%) nodules were read as follicular neoplasm/suspicious for follicular neoplasm (Bethesda 4). Nondiagnostic, suspicious for malignancy, or frankly malignant cytological results (Bethesda 1, Bethesda 5, and Bethesda 6) were not encountered (Table 1).

The Afirma GEC® gene expression classifier was requested in eleven nodules with Bethesda 3 and three nodules with Bethesda 4 cytology. The GEC result was “suspicious” in all eleven Bethesda 3 nodules and in two of the three Bethesda 4 nodules. The Afirma GEC “benign” Bethesda 4 nodule was removed due to its size (6.9 cm) and suspicious sonographic features (Patient 4 in Table 1). Two nodule aspirates (Patients 1 and 6, Table 1) underwent ThyroSeq 2.1® mutational analysis and both exhibited DNA alterations, including a PAX8/PPAR gamma translocation and an NRAS mutation with an allelic frequency of 4%.

Twenty patients were treated with total thyroidectomy (one with central compartment lymphadenectomy) and 17 patients underwent lobectomy. Four patients who had an initial lobectomy underwent a completion thyroidectomy and 2 of these 4 patients had microscopic follicular variant of papillary cancer in the lobe opposite to the previously removed noninvasive EFVPTC tumor. Permanent postsurgical hypoparathyroidism and vocal cord dysfunction were not encountered in any of the 37 patients.

On histologic examination, 13 out of 37 (35%) thyroidectomy specimens were found to contain additional incidental malignancies. These included 10 patients with incidental subcentimeter papillary thyroid cancers (PTCs) or follicular variant of papillary cancers (FVPTCs) (one with a single positive central compartment LN, Patient 3, Table 1), three patients with macroscopic PTC or FVPTCs (one with positive central LNs, Patient 34, Table 1). Five of the 13 synchronous malignancies noted in our noninvasive EFVPTC patients (38%) were in the lobe opposite to the EFVPTC tumor.

Two of the 37 patient specimens (5%) contained one or more malignant lymph nodes with subcentimeter, noninvasive tumor deposits. In the first patient (Patient 3, Table 1) the tumor responsible for a single right paratracheal lymph node microscopic metastasis was suspected to be a synchronous right lobe subcentimeter (contralateral from the left sided noninvasive EFVPTC) classical papillary thyroid cancer. In the second patient with an 0.8 cm isthmic noninvasive EFVPTC (Patient 34, Table 1), the tumor responsible for the microscopic lymph node metastases was a left lobe 1.5 cm classical papillary thyroid cancer (4 of 9 lymph nodes were positive for microscopic metastases in the adjacent left thyroid bed).

Two of the 37 patients received adjuvant radioactive iodine therapy postoperatively. Patient 34 received adjuvant radioactive iodine for her 1.5 cm classical papillary cancer with 4 left paratracheal metastatic lymph nodes. Another patient (Patient 1, Table 1) with a 6.1 cm noninvasive EFVPTC tumor received ablative radioiodine therapy at another institution based on tumor size alone.

## 4. Discussion

We describe the clinical characteristics of 37 tumors from 37 patients with noninvasive encapsulated follicular variant of papillary thyroid cancer (EFVPTC) culled from a community-based endocrine surgery registry from January 2013 through September 2016.

Our noninvasive EFVPTC patients were relatively young (mean age 44) and predominately female with nodules that, on ultrasound, were large (3.1 cm on average), hypo- to isoechoic, and mildly hypervascular with crisp boundaries in all 37 cases. Highly suspicious sonographic features such as microscopic calcifications or infiltrative borders were not seen in any of these lesions. Noninvasive EFVPTC nodules typically yielded indeterminate cytology results on fine needle aspiration biopsy (73% Bethesda Classification 3 or 4) with 12 of the 13 (92%) who underwent Afirma GEC testing classified as “suspicious.” Although we did not routinely perform mutational testing at our center from 1/2013 to 9/2016, two of our patients were found at outside institutions to have DNA alterations including a PAX8/PPAR gamma translocation and a low level NRAS mutation affecting 8% of thyrocytes, corresponding to an allelic frequency of 4%. Although we did not recut surgical specimens, our noninvasive EFVPTC patients' clinical, ultrasound, cytology, and molecular characteristics appear to resemble those of the NIFTP tumor patients described by Nikiforov et al. [4] in 2016.

Interestingly, in our patient group, molecular markers influenced surgical decision-making in 16 of the 17 patients in whom molecular testing was performed, while the other twenty patients had surgical decision-making driven by other factors such as family history, clinical symptoms, and, most importantly, ultrasound findings. Cytology evaluation was less helpful as all our patients who underwent fine needle aspiration biopsy (FNA) fell into Bethesda categories 2 (Benign), 3 (AUS/FLUS), or 4 (FN/SFN).

Twenty out of 37 of our noninvasive EFVPTC patients (54%) underwent total thyroidectomy due to primary tumor size, tumor appearance, or ultrasound demonstrable contralateral disease. One of these twenty patients underwent a total thyroidectomy and a central lymph node dissection due to suspected central lymph involvement and subsequently received postoperative radioiodine therapy for remnant ablation when 4 central lymph nodes were found to contain microscopic cancer on pathologic examination with the largest nodal deposit measuring 0.4 cm (Patient 34, Table 1). Interestingly, this patient had a synchronous nonencapsulated 1.5 cm conventional papillary cancer in the adjacent left thyroid lobe, remote from the primary isthmic noninvasive EFVPTC on pathologic evaluation. A second patient with an incidentally discovered single paratracheal lymph node containing a microscopic metastasis was noted to have subcentimeter conventional papillary cancer in the lobe ipsilateral to the lymph node but contralateral to the noninvasive EFVPTC (Patient 34, Table 1) and was not treated with RAI.

At the time of this writing, we have not seen a clinical recurrence in any of our 37 noninvasive EFVPTC patients, but our average length of follow-up of 18 months is far too short to allow a definitive conclusion of benign clinical behavior.

Thirteen of our 37 noninvasive EFVPTC patients (35%) had synchronous thyroid cancers elsewhere in the thyroid gland. This is a higher than expected rate of thyroidectomy-detected occult microscopic carcinoma with the typical reported rate approximating 10% [7–10]. Although incidental thyroid microscopic carcinoma rates as high as 35.6% have been reported at autopsy in Finland by Harach et al. [11], many researchers view the Finnish data point as an outlier. At this point, speculation on whether the thyroid gland containing NIFTP-like tumors is a particularly fertile microenvironment for thyroid cancer generation is premature, but our data suggest that the entire central and lateral neck should be carefully imaged with ultrasound in patients with suspected noninvasive encapsulated follicular variant of papillary cancers. Although our preliminary follow-up data indicates that our patients with noninvasive EFVPTC tumors behave in a benign fashion, other synchronous thyroid tumors in these patients may not. Specifically, two of our 37 patients demonstrated remote thyroid bed metastatic lymph nodes from more aggressive thyroid cancers elsewhere in their thyroid glands (Table 1, patients 3 and 34). Thus, the frequency and character of synchronous thyroid cancer development in patients with noninvasive EFVPTCs are an important topic for further prospective clinical investigation.

**Figure 1:**
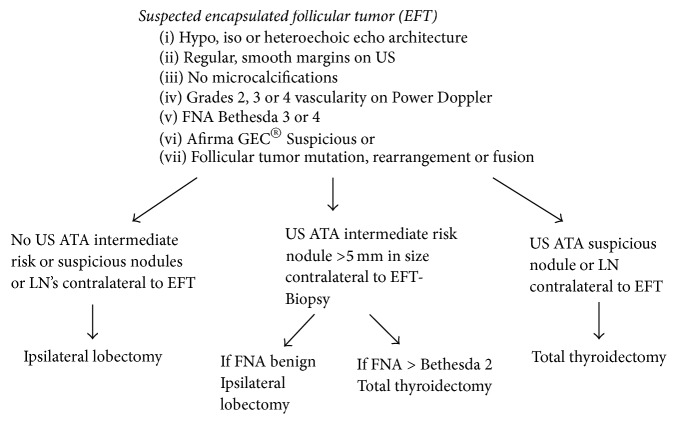
Algorithm for surgical decision-making with suspected encapsulated follicular tumors.

In the current endocrine surgical practice environment we lack prospective studies that provide guidance for the surgical management of patients with suspected noninvasive EFVPTCs and/or NIFTP tumors (collectively referred to as “encapsulated follicular tumors”). Our current approach is to assess all patients referred for thyroid nodule evaluation for characteristic ultrasound and fine needle aspiration features of encapsulated follicular tumors which include (1) nonhyperechoic lesion on ultrasound, (2) smooth, uniform, and uninterrupted margins, (3) no microscopic calcifications, (4) Bethesda 3 or 4 cytology, and (5) either a suspicious Afirma GEC or a mutation panel demonstrating a follicular tumor mutation, rearrangement, or fusion (specifically, no BRAF V600, RET/PTC, RET, TERT, TP53, or PTEN mutations, rearrangements, or fusions, all associated with other more aggressive thyroid cancer subtypes) (Figure 1). In suspected encapsulated follicular tumor patients, we then carefully evaluate the contralateral thyroid lobe and central compartment for additional ultrasound and fine needle aspiration findings that could potentially change the surgical recommendation from lobectomy to thyroidectomy or to thyroidectomy with central or lateral neck dissection. We currently recommend routine fine needle aspiration biopsy (FNA) of all American Thyroid Association (ATA) intermediate risk hypoechoic, solid nodules [12] greater than 5 mm in diameter in the contralateral thyroid lobes of these patients (opposite the “encapsulated follicular tumor” lobe) to facilitate the surgical choice between lobectomy and thyroidectomy [12]. Our proposed scheme for surgical decision-making is outlined in Figure 1. If the fine needle aspiration biopsy result for a contralateral hypoechoic, smooth capsuled nodule above 5 mm in size returns at any Bethesda level above 2 (Bethesda 3–6), we plan for a total thyroidectomy. If, at ultrasound evaluation, we discover contralateral high risk lesions or suspicious lymph nodes, we plan for a total thyroidectomy, foregoing additional fine needle aspiration (FNA) biopsy. If the contralateral neck is pristine by ultrasound or if contralateral FNA biopsy of ATA intermediate ultrasound risk nodules is benign, we plan for ipsilateral (encapsulated follicular tumor affected lobe only) thyroid lobectomy. We emphasize that this algorithm is a “proposed” surgical management scheme with no prospective data to support it. However based on our finding of a 35% prevalence of multifocal tumor occurrence in patients with noninvasive encapsulated follicular variant of papillary cancers, we feel that aggressive ultrasound and FNA biopsy evaluation of contralateral neck abnormalities is essential in these patients until prospective studies emerge. In addition, when dealing with suspected encapsulated follicular tumors intraoperatively, we encourage thyroid surgeons to seek frozen section guidance on any suspicious lymph nodes discovered during the operative procedure.

In conclusion, because of a high risk of tumor multifocality, careful preoperative neck ultrasound and appropriate use of fine needle aspiration biopsy are essential in the management of suspected noninvasive EFVPTC tumors. The extent of surgery should be guided by ultrasound findings, biopsy results and clinical judgement. Molecular testing with Afirma GEC is often “suspicious” in noninvasive encapsulated follicular variant of papillary thyroid cancers and should lead to ipsilateral lobectomy and isthmusectomy in the absence of historical, clinical, neck ultrasound or fine needle aspiration biopsy findings elsewhere suggesting metastatic lymph node involvement or synchronous thyroid malignancy [13].

## Figures and Tables

**Table 1:** Demographics, US, surgery and pathology results in 37 noninvasive EFVPTC patients.

Patient	Gender	Age	EFVPTC size (cm)	Echo type	Cytology	Afirma GEC	Operation	Nodes	Other cancer
1^*∗*^ ^*∗∗*^	F	34	6.1	Iso	FN/SFN		TT	0/1	No
2	M	52	5.6	Hetero	Benign		LHT, RHT	0/1	No
3	F	31	4.7	Iso	Benign		TT	1/1^*∗∗∗∗*^	MicroPTC-c
4	F	69	6.9	Hypo	FN/SFN	Benign	TT	0/0	No
5	F	36	4.5	Hypo			TT	0/0	MicroPTC-c
6^*∗∗∗*^	F	27	4.2	Hetero	FLUS/AUS		LHT	0/1	No
7	F	55	5.8	Iso			LHT	0/0	No
8	M	30	4.1	Iso	FLUS/AUS	Suspicious	RHT	0/0	No
9	F	51	3.9	Hetero	FLUS/AUS	Suspicious	TT	0/1	MicroFvPTC
10	M	56	3.6	Hetero	FLUS/AUS	Suspicious	LHT	0/3	MicroPTC
11	F	41	4.4	Iso	FN/SFN	Suspicious	RHT	0/0	No
12	F	50	3.1	Iso			LHT	0/2	No
13	F	38	3.8	Iso			LHT	0/1	No
14	F	30	2.8	Iso	FN/SFN		LHT	0/1	No
15	F	35	2.6	Hetero	Benign		TT	0/1	No
16	F	21	3.15	Hetero	FN/SFN		RHT	0/1	No
17	F	50	2.4	Hypo	Benign		TT	0/3	MicroPTC
18	F	60	3	Iso	FLUS/AUS	Suspicious	TT	0/0	MicroFvPTC
19	F	46	1.4	Hypo	FLUS/AUS	Suspicious	RHT	0/1	No
20	M	38	3	Iso	FLUS/AUS	Suspicious	TT	0/2	MicroPTC
21	F	32	3.3	Iso	FN/SFN	Suspicious	TT	0/0	No
22	M	53	2.5	Iso	FN/SFN		RHT	0/0	No
23	F	16	2.7	Iso	Benign		LHT, RHT	0/1	MicroFvPTC-c
24	F	37	2.63	Hypo	FLUS/AUS		TT	0/3	No
25	F	53	1.9	Hypo			LHT, RHT	0/1	2.1 cm FVPTC and microFVPTC-c
26	F	56	2.53	Hetero	Benign		RHT, LHT	0/1	MicroPTC
27	F	55	1.6	Hypo	FLUS/AUS		TT	0/1	No
28	M	39	2.1	Iso	FLUS/AUS	Suspicious	LHT	0/1	No
29	F	40	1.3	Hypo	FLUS/AUS	Suspicious	TT	0/0	No
30	M	64	1.6	Hypo	Benign		TT	0/0	No
31	F	58	1.3	Iso	FLUS/AUS	Suspicious	LHT	0/1	MicroPTCs
32	F	55	1.1	Iso	FLUS/AUS	Suspicious	TT	0/0	No
33	F	37	0.8	Iso			TT	0/4	No
34^*∗*^	F	37	1.3	Iso			TT + CND	4/9^*∗∗∗∗*^	1.5 cm PTC-c
35	F	45	0.9	Iso	FLUS/AUS	Suspicious	TT	0/0	2 cm FvPTC
36	F	32	4	Iso	FN/SFN		TT	0/0	No
37	M	56	4.3	Iso	Benign		TT	0/0	No

^*∗*^Patient treated with radioiodine, ^*∗∗*^PAX8/PPAR gamma translocation, ^*∗∗∗*^NRAS mutation, ^*∗∗∗∗*^nodal micrometastases, and -c contralateral lobe from encapsulated EFVPTC tumor.
